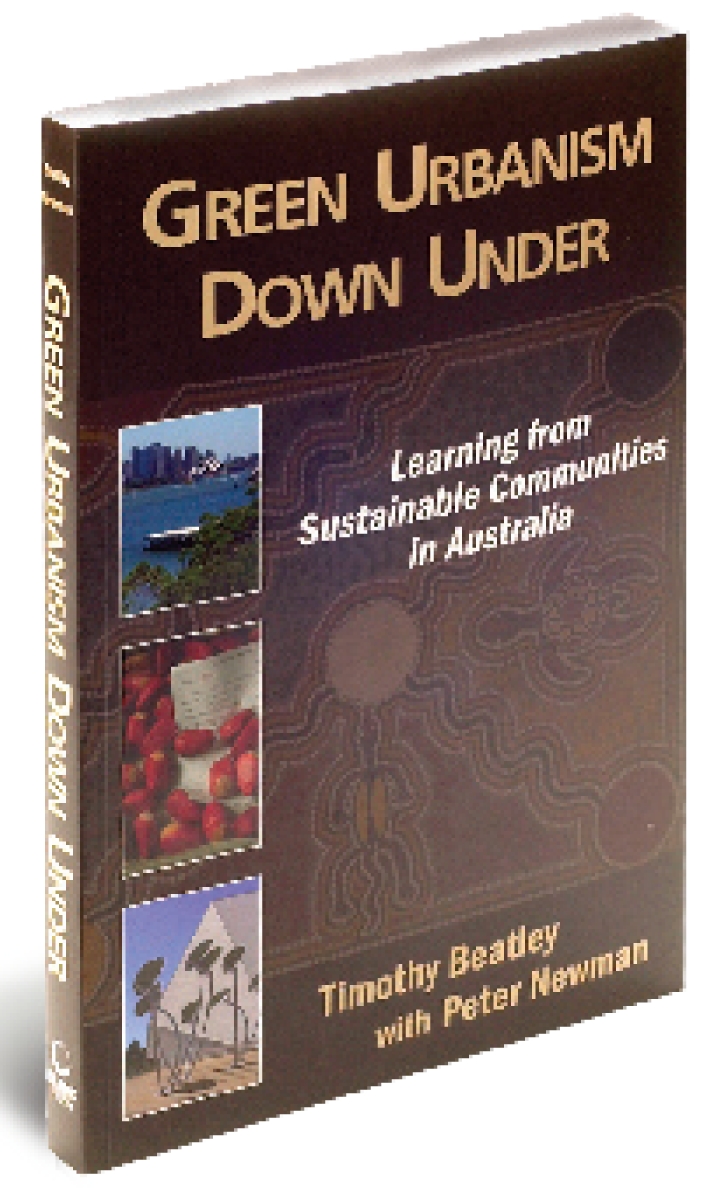# Resilient, Green… and Healthy

**Published:** 2009-07

**Authors:** Howard Frumkin

**Affiliations:** Howard Frumkin is director of the National Center for Environmental Health and Agency for Toxic Substances and Disease Registry at the U.S. Centers for Disease Control and Prevention

The environmental health field has been swept by major changes in recent years. One was the rediscovery of the “built environment” as a key determinant of human health. Community design—including land use and transportation decisions, but extending to such arenas as energy, housing, and food—affects outcomes as diverse as cardiovascular health, respiratory heath, cancer, injury risk, and social capital.

A second major advance has been the growing appreciation of global change as a fundamental driver of human health. Climate change is perhaps the best recognized example, but other global changes—peak petroleum, biodiversity loss, land use changes, and resource depletion—also loom large.

The two books reviewed here combine both domains. They describe how global changes will affect what is now the predominant human habitat: the city. But the books do more than describe; they brim with recommendations for how cities can become more resilient to global changes, more sustainable, and implicitly (for health is rarely mentioned in either book) more healthy.

The books are the product of a scholarly dream team. Timothy Beatley is professor of sustainable communities at the University of Virginia, and Peter Newman is professor of sustainability at Curtin University in Australia, both leading authorities on sustainable community design. Heather Boyer, co-author of *Resilient Cities*, is a seasoned editor at Island Press, the preeminent environmental publisher.

*Resilient Cities* is a succinct, wide-reaching, and readable overview, with several important attributes. First, it considers two major global trends, climate change and peak petroleum, jointly; too many recent analyses have focused on one or the other of these, although they will clearly emerge in tandem. Second, it is suffused with hope, a deliberate counterpoint to the gloom and doom typical of so much global change discourse. Third, it is grounded in the concept of resilience—“the capacity of a system to absorb disturbance and still retain its basic function and structure”—as applied to cities. Finally, the authors recognize that resiliency is not simply a technical challenge; it requires civic commitment to long-term planning, participatory democracy, cooperation, and multisectoral partnership.

The book posits four possible scenarios for resource-intensive cities: collapse, reversion to rural form, division, or resiliency. Collapse is conceivable but certainly not desirable. The “ruralized city” has been advocated by some peak oil commentators, who point out that local agricultural production would both utilize idle land in sprawling communities and reduce the need for long-distance transport of food. But Newman, Beatley, and Boyer dismiss the idea except on a limited scale, arguing that agriculture “is not the primary function of a city.” The divided city is a stratified and polarized place, with gated enclaves for the well off and various degrees of squalor, danger, and hopelessness for the less fortunate. Given these scenarios, it is no surprise that the authors vote for resiliency: green buildings, reduced energy demand, reliance on renewable fuel sources, walkable neighborhoods, more localized production of goods, and other hallmarks of sustainability.

Following descriptions of climate change and peak petroleum and an inventory of the options for responding, *Resilient Cities* moves to compelling chapters on the built environment and on transportation. These are practical, sensible chapters, generously endowed with examples from around the world: urban gardens in Vancouver, biogas from wastewater in Stockholm, tree planting in Sacramento, solar energy in Toronto and Austin, wind turbines in Honolulu, energy-efficient building standards in Freiburg, congestion taxes in London. We learn of aspirational programs such as carbon-neutral cities (Malmö, Newcastle, and Adelaide) and zero-energy developments (the Beddington Zero Energy Development, or BedZED, in the London borough of Sutton). One senses that these are not abstractions or impossibilities; they are practical, affordable, and readily achievable. There is reason for hope.

*Green Urbanism Down Under* extends this discussion. Timothy Beatley spent 6 months living and traveling in Australia, and this book is the result. He sees Australia as similar in many ways to the United States: It is extensively urbanized, it is very resource- and energy-consumptive, it relies heavily on fossil fuels despite having extensive renewable energy resources, it is automobile-dependent, it has a large per capita ecologic footprint, and it has a constitutional democracy. Beatley persuasively maintains that Australia has many lessons to offer the United States.

The longest chapter is a detailed account of sustainability practices in Australia’s five major cities—Sydney, Melbourne, Brisbane, Adelaide, and Perth. They are far advanced, and there is much to learn from them. The next chapter expands the focus to entire bioregions, the following chapter discusses ways in which the sense of place is strengthened in localities across Australia, and the subsequent chapter focuses on natural amenities (the “bush,” in Australian parlance) in cities. The penultimate chapter analyzes planning practices at the regional and state levels, and the final chapter discusses the relevance of the book to the United States.

Readers who care about sustainability and urban form will greatly enjoy these books. Readers who care about environmental change—especially emerging long-term threats such as climate change and peak petroleum—will also learn much from reading these books. But readers of this journal, who care about the specifics of human health effects, will not be fully satisfied. Both books overlook almost entirely the health issues inherent in climate change and peak petroleum. Health threats are not mentioned, and though the authors certainly appreciate the value of co-benefits (“a more localized approach will save oil, minimize greenhouse gas emissions, and help create better local economies and communities”), they unaccountably omit health benefits from their discussion of resiliency.

*Resilient Cities* and *Green Urbanism Down Under* are delightful books. Together they offer a positive, inspiring view of how communities can be designed not only to withstand global changes, but to thrive by adopting green practices. Although the books offer little direct information on health, they provide an invaluable framework for considering the public health approach to both climate change and peak petroleum.

## Figures and Tables

**Figure:**
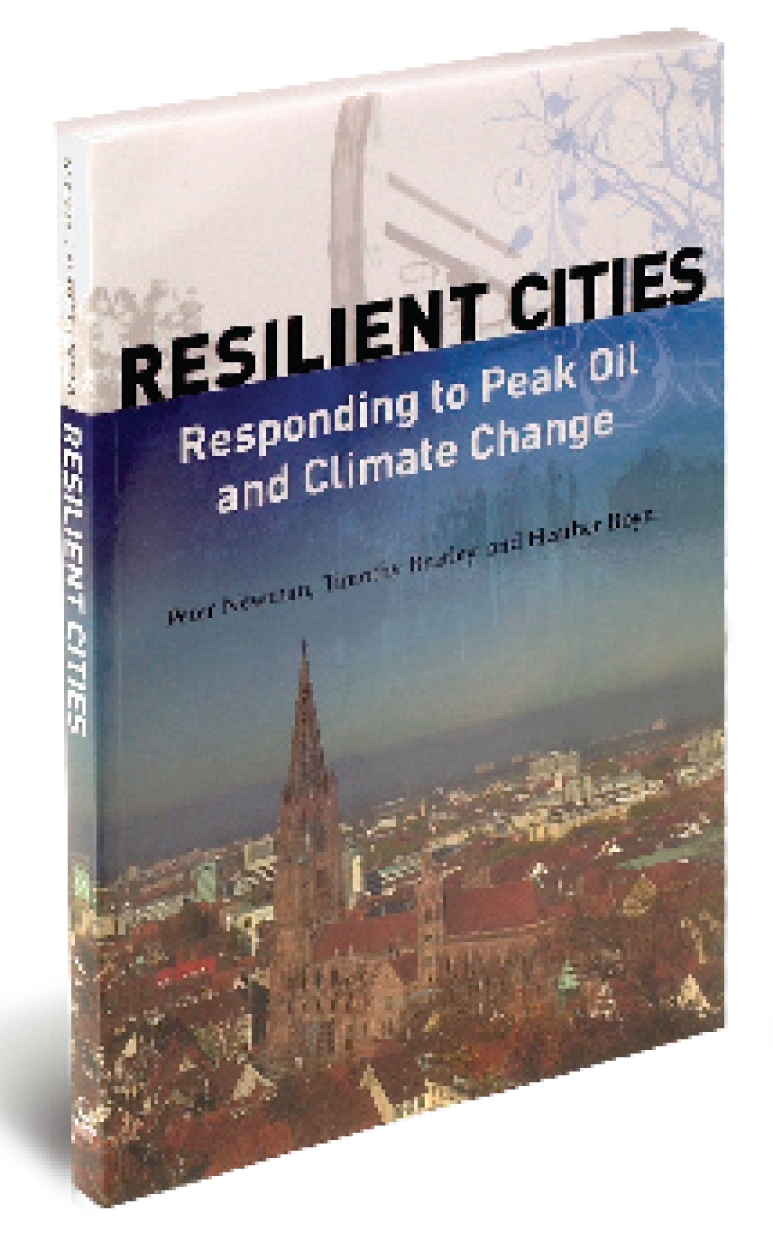


**Figure:**